# Resveratrol Maintains Lipid Metabolism Homeostasis via One of the Mechanisms Associated with the Key Circadian Regulator Bmal1

**DOI:** 10.3390/molecules24162916

**Published:** 2019-08-12

**Authors:** Jing Li, Liping Wei, Caicai Zhao, Junyi Li, Zhigang Liu, Min Zhang, Yutang Wang

**Affiliations:** 1 Beijing Advanced Innovation Center for Food Nutrition and Human Health, Beijing Technology and Business University, Beijing 100089, China; 2 College of Food Science and Engineering, Northwest A&F University, Yangling, Shaanxi 712100, China

**Keywords:** resveratrol, Bmal1, circadian rhythm, glycolipid metabolism, nonalcoholic fatty liver disease

## Abstract

Resveratrol (RES) possesses anti-inflammatory and anti-oxidant activities, and it can prevent liver lipid metabolism disorders in obese and diabetic individuals. This study elucidated the mechanisms of brain and muscle Arnt-like protein-1 (Bmal1) in the protective effects of RES against liver lipid metabolism disorders. The results indicated that RES ameliorated free fatty acid (FFA)-induced (oleic acid (OA): palmitic acid (PA) = 2:1) glycolipid metabolic disorders in hepatocytes. Simultaneously, RES partially reverted the relatively shallow daily oscillations of FFA-induced circadian clock gene transcription and protein expression in HepG2 cells. RES also attenuated FFA-triggered reactive oxygen species (ROS) secretion and restored mitochondrial membrane potential consumption, as well as the restoration of mitochondrial respiratory complex expression. This study provides compelling evidence that RES controls intracellular lipid metabolic imbalance in a Bmal1-dependent manner. Overall, RES may serve as a promising natural nutraceutical for the regulation of lipid metabolic disorders relevant to the circadian clock.

## 1. Introduction

In recent years, the incidence of obesity has sharply increased, posing a great threat to human health [1]. Obesity can lead to disorders of glycolipid metabolism and a series of metabolic impairments, such as insulin resistance (IR), nonalcoholic fatty liver disease (NAFLD), type 2 diabetes mellitus, and atherosclerosis [2]. An unhealthy lifestyle, such as excessive consumption of energy-intensive food and reduced physical activity, may be an important factor in metabolic disorders [1]. Disorders of lipid metabolism in the liver caused by obesity can lead to NAFLD, a common liver disease that causes extensive liver damage and eventually develops into nonalcoholic steatohepatitis, which manifests by excessive accumulation of triacylglycerols in the liver, inflammation, and liver function damage [3,4]. IR, a declined response of the peripheral tissues to insulin action, may contribute to the development of fatty liver by impairing the ability of insulin to inhibit lipolysis, leading to an increased delivery of free fatty acids (FFAs) to the liver [5,6,7].

Epidemiological studies have shown that the circadian rhythm is closely related to metabolic homeostasis [8]. In a great range of species, the circadian clock has evolved to adapt to the Earth’s rotation [9]. The circadian rhythm is a 24 h internal oscillator that plays a crucial role in coordinating internal behavioral and physiological rhythms [10]. In mammals, the circadian rhythm system consists of the central circadian clock located in the hypothalamic suprachiasmatic nuclei (SCN) and the peripheral circadian clock located in the peripheral tissues and organs, such as the liver and heart, present in almost all cells of the body [11,12,13]. At the molecular level, circadian rhythm maintenance relies on an interlocked transcription–translation feedback loop composed of a series of clock proteins, including circadian locomotor output cycles kaput (Clock), brain and muscle Arnt-like protein-1 (Bmal1), period genes (Per), and cryptochrome genes (Cry) [14,15]. In the peripheral clock systems, the circadian rhythms are closely associated with various physiological processes, such as lipid, glucose, and cholesterol metabolism [16].

Epidemiological studies have shown that circadian misalignment can contribute to a wide variety of metabolic impairments, including hypertension, obesity, and IR, that seriously threaten human health [17]. Circadian gene clock mutant mice exhibit an extremely decreased diurnal feeding rhythm, hyperphagia, and obesity, and develop into a metabolic syndrome of hyperlipidemia, hepatic steatosis, hyperglycemia, and hypoinsulinemia [18]. Metabolic systems may feed back into the circadian system and regulate circadian gene expression and behavior [19]. A high-fat diet (HFD) in mice leads to an altered locomotor activity rhythm and changes in the expression and cycling of core circadian clock genes, as well as nuclear receptors that regulate clock transcription factors in the liver [20]. Additionally, we have recently reported that FFA or palmitic acid (PA) treatment in mouse primary hepatocytes and HepG2 cells markedly weaken mRNA and protein expression levels of Clock and Bmal1 [21,22]. Many regulators and enzymes of lipid biosynthesis and catabolism are dominated via the circadian gene network [13]. The evidence above indicates the unshakable role of the circadian clock in maintaining glycolipid metabolism homeostasis.

Bioactive components in natural foods, such as proanthocyanidins, (-)-Epigallocatechin-3-gallate (EGCG), chicoric acid, and nobiletin, can manipulate circadian rhythms to ameliorate metabolic syndrome [21,22,23]. Resveratrol (RES, 3,5,4′-trihydroxystilbene), a natural product derived from grapes, has various health benefits, including anti-inflammatory, anti-oxidant, and anti-mutation properties [24]. In addition, RES can maintain lipid metabolism homeostasis and effectively alleviate IR [25]. Sun et al. revealed that RES abolished HFD-induced circadian desynchrony and alleviated the abnormal lipid metabolism in C57BL/6J mice [26]. However, whether circadian clock mechanisms are involved in the regulation of FFA-induced glycolipid metabolism disorder by RES remains unknown.

The present study aims to reveal the intervention effect of resveratrol on the imbalance of hepatocyte lipid metabolism and the mechanism of participation of the circadian clock. Moreover, we also explored the effects of resveratrol on FFA-induced oxidative stress and mitochondrial dysfunction in hepatocytes. It is our hope that the present work can provide a theoretical basis and new insights for the extended application of RES.

## 2. Results

### 2.1. Intervention Effect of RES on Morphology Changes and Intracellular Lipid Accumulation under High Fatty Acid Conditions

**Figure 1:**
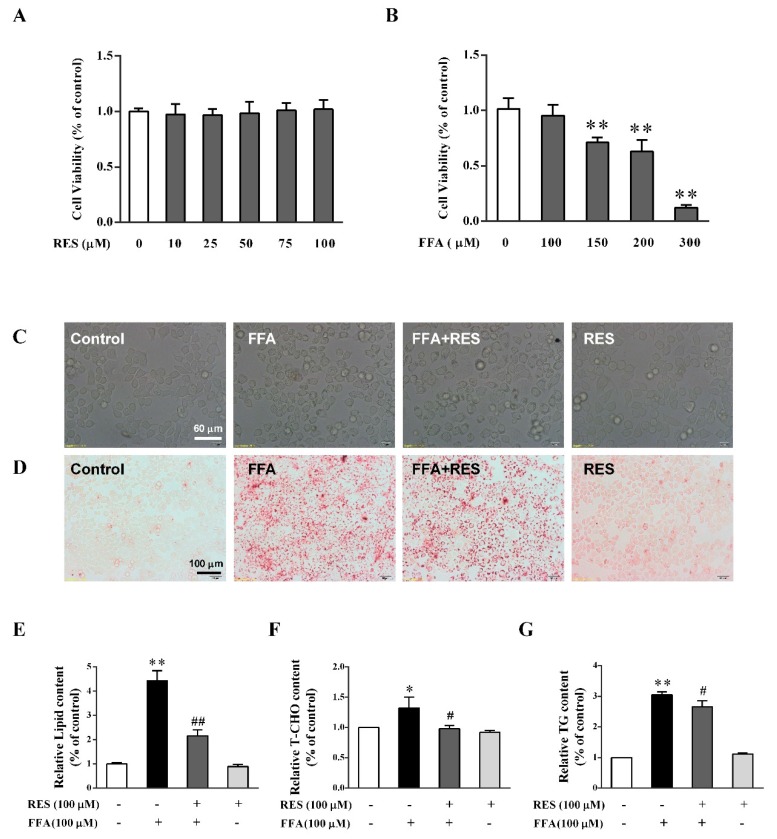
Intervention effect of resveratrol (RES) on morphological changes and intracellular lipid accumulation under high-fatty-acid conditions. HepG2 cells were treated with different concentrations of (**A**) RES (0–100 µM) for 6 h and (**B**) Free fatty acid (FFA) (0–300 µM) for 24 h. Cell proliferation was measured by 3-(4,5-dimethyl-2-thiazolyl)-2,5-diphenyl-2-H-tetrazolium bromide (MTT) assay. (**C**) HepG2 cells were pretreated with/without RES (100 µM) for 6 h and then cultured with/without FFA (100 µM) with 0.1% bovine serum albumin (BSA) for 24 h. Morphological changes of HepG2 cells were determined using an inverted microscope (×400). Lipid accumulation of HepG2 cells was determined by Oil Red O staining, qualitatively analyzed using an inverted microscope (×200) (**D**), and quantitatively analyzed using a microplate reader (492 nm) (**E**). Relative cellular levels of (**F**) total cholesterol (T-CHO) and (**G**) triglycerides (TG) were detected using corresponding kits. Data were presented as the mean ± SD, *n* ≥ 3. (∗) *p* < 0.05 and (∗∗) *p* < 0.01, versus control group; (#) *p* < 0.05 and (##) *p* < 0.01, versus FFA group.

To evaluate the protective effects of RES on cell viability and proliferation, HepG2 cells were treated with various concentrations of RES (0–100 µM) for 6 h or FFA (0–100 µM) for 24 h. The results showed that 100 µM RES and 100 µM FFA had no significant toxic effects on HepG2 cells (Figure 1A,B). However, the optical microscopic observation indicated that cell morphology changed to a spheroid shape after FFA exposure, but this change was partially reversed by RES (Figure 1C).

As shown in Figure 1D,E, FFA (100 µM) notably increased the intensity of Oil Red O staining in cells, indicating that it can be used as a potential model to stimulate metabolic disorders on hepatocytes. However, the results showed that RES pretreatment could decrease the intensity of Oil Red O staining (Figure 1D,E) triggered by FFA. Moreover, FFA dramatically increased the accumulation of triglycerides (TG) and total cholesterol (T-CHO) in HepG2 cells, which was significantly reverted by RES (Figure 1F,G). In short, RES may be beneficial to physiological health by protecting against FFA-triggered lipotoxicity in HepG2 cells.

### 2.2. RES Ameliorates FFA-Triggered Hepatic Glucose Metabolism and Lipid Metabolism Unbalance

**Figure 2:**
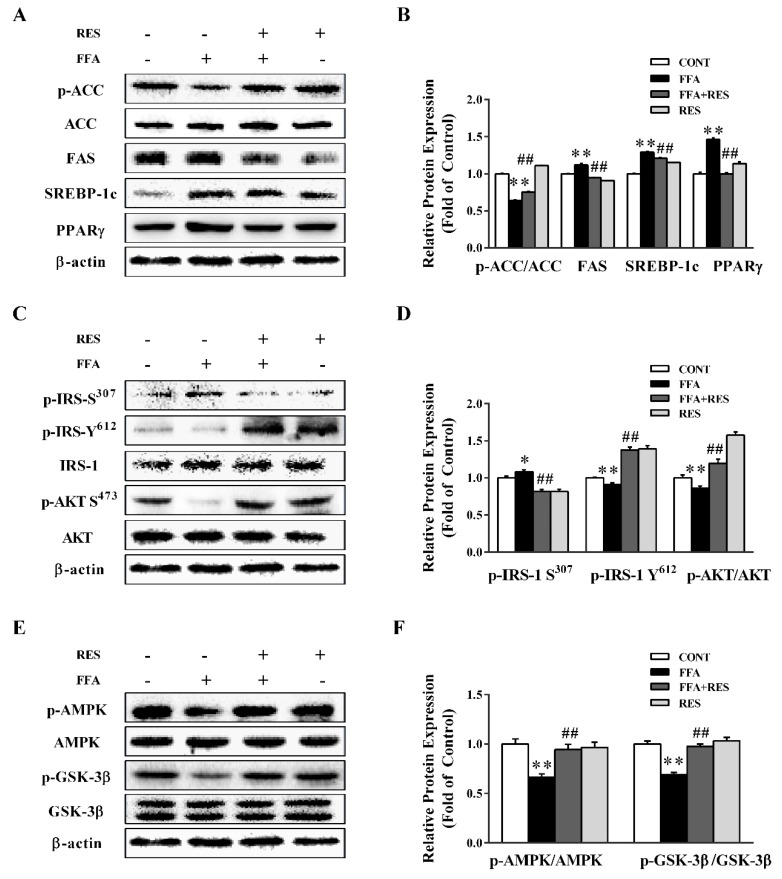
RES ameliorates FFA-triggered hepatic glucolipid metabolism disorders in HepG2 cells. HepG2 cells were pretreated with RES (100 µM) for 6 h and with FFA (100 µM) for 24 h. (**A**) The effect of RES on FFA-induced abnormal lipid metabolism was determined by western blot analysis. phospho-acetyl-CoA carboxylase (p-ACC), total acetyl-CoA carboxylase (ACC), fatty acid synthase (FAS), sterol regulatory element-binding protein 1c (SREBP-1c), and peroxisome proliferator activated receptor gamma (PPARγ) were detected in HepG2 cells, and β-actin was used as a loading control. (**C**) The effect of RES on FFA-induced insulin signaling changes was determined by western blot analysis. p-IRS-1 Tyr^612^, p-IRS-1 Ser^307^, total insulin receptor substrate 1 (IRS-1), p-AKT Ser^473^, and total protein kinase B (AKT) were detected in cells, and β-actin was used as a loading control. (**E**) Representative western blot of p-AMPK, p-GSK3β, total AMP-activated protein kinase (AMPK), and total GSK3β after treatment with RES and FFA in HepG2 cells. (**B**), (**D**), and (**F**) are the densitometric analysis of the blots shown in (**A**), (**C**), and (**E**), respectively. Data were presented as the mean ± SD, *n* ≥ 3. (∗) *p* < 0.05 and (∗∗) *p* < 0.01, versus control group; (#) *p* < 0.05 and (##) *p* < 0.01, versus FFA group.

To investigate the role of RES in modulating lipid metabolism and IR in HepG2 cells and primary hepatocytes, lipid metabolism-related proteins and the phosphorylation of insulin receptor substrate 1 (IRS-1) and downstream pathways were determined. As shown in Figure 2A,B and Figure S1E,F, FFA notably decreased acetyl-CoA carboxylase (ACC) phosphorylation and stimulated the expression of key enzymes of *de novo* lipogenesis, including fatty acid synthase (FAS), sterol regulatory element-binding protein 1c (SREBP-1c), and peroxisome proliferator activated receptor gamma (PPARγ). Interestingly, RES pretreatment substantially restored the FFA-induced changes. As shown in Figure 2C,D, FFA treatment dramatically increased IRS-1 (Ser 307) phosphorylation to 108.3% and reduced IRS-1 (Tyr 612) phosphorylation to 91.1%, which was significantly reverted by RES pretreatment to 81.5% and 137.7%, respectively. Downstream of IRS, FFA treatment showed a notable decrease in protein kinase B (AKT) phosphorylation, which was partially counteracted by RES.

As presented in Figure 2E,F and Figure S1C,D, AMP-activated protein kinase (AMPK) phosphorylation was down-regulated by FFA and partially reversed by RES pretreatment. Similarly, RES remarkably improved the activation of glycogen synthase kinase-3 (GSK-3β), which was inhibited by FFA (Figure 2E,F). Collectively, RES significantly attenuated lipid metabolism imbalance and IR induced by FFA.

### 2.3. RES Attenuates FFA-Triggered Oxidative Stress and Mitochondrial Dysfunction in Hepatic HepG2 Cells.

**Figure 3:**
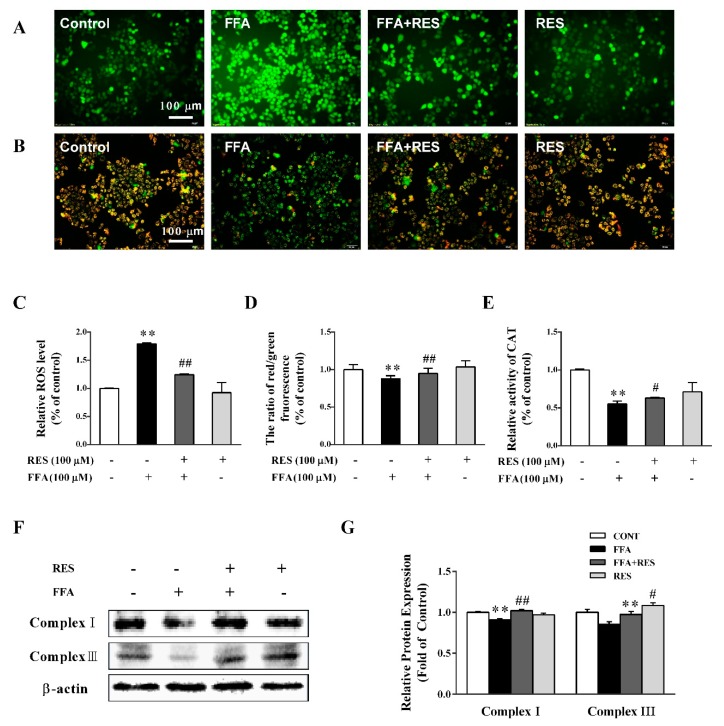
RES attenuates FFA-triggered oxidative stress and mitochondrial dysfunction in hepatic HepG2 cells. HepG2 cells were pretreated with RES (100 µM) for 6 h and with FFA (100 µM) for 24 h. Then, the cells were stained with 2′,7′-dichlorodihydrofluorescein diacetate (DCFH-DA) to detect intracellular reactive oxygen species (ROS) levels, (**A**) and (**C**). (**B**) and (**D**) mitochondrial membrane potential (MMP) was examined by JC-1 staining (×200). (**E**) Intracellular catalase (CAT) activity. (**F**) Expression levels of mitochondrial complexes, complex I and complex III, in total cell lysates using western blot analysis. β-actin was used as a loading control. (**G**) Densitometric analysis of the blots shown in (**F**). Data were presented as the mean ± SD, *n* ≥ 3. (∗) *p* < 0.05 and (∗∗) *p* < 0.01, versus control group; (#) *p* < 0.05 and (##) *p* < 0.01, versus FFA group.

The high intracellular reactive oxygen species (ROS) level (Figure 3A,C) induced by FFA treatment was eliminated by RES in HepG2 cells. As illustrated in Figure 3B,D, the FFA-elicited decrease in mitochondrial membrane potential (MMP), both qualitatively and quantitatively, which is an early sign of mitochondrial dysfunction, was partially reverted by RES pretreatment. RES pretreatment markedly reversed the abnormal changes of cellular catalase (CAT) activity induced by FFA (Figure 3E). Consistently, the protein expression levels of mitochondrial respiratory chain complex I (NADPH-diaphorase) and III (cytochrome reductase) were significantly inhibited by FFA exposure but partially restored by RES (Figure 3F,G).

**Figure 4:**
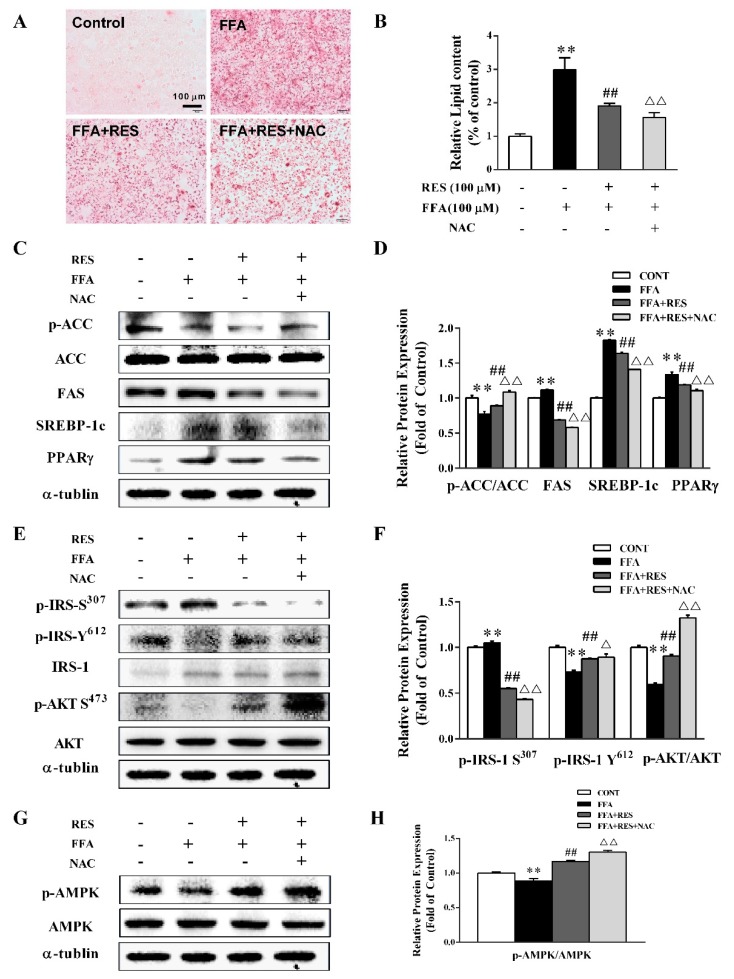
RES restores FFA-induced impairment of hepatic lipid metabolism via the elimination of ROS. HepG2 cells were preincubated for 30 min with/without N-acetylcysteine (NAC) (10 µM) and then pretreated with/without RES (100 µM) for 6 h and with FFA (100 µM) for 24 h. (**A**) Lipid accumulation of HepG2 cells was measured by Oil Red O staining and qualitative analysis using an inverted microscope (×200). (**B**) Quantitative analysis shown in (A)using a microplate reader (492 nm). (**C**) The expression levels of p-ACC, total ACC, FAS, SREBP-1c, and PPARγ was detected in cells by western blot analysis, and α-tubulin was used as a loading control. (**E**) The effect of RES on FFA-induced insulin signaling changes was determined by western blot analysis. p-IRS-1 Tyr^612^, p-IRS-1 Ser^307^, total IRS-1, p-AKT Ser^473^, and total AKT were detected in cells, IRS-1, AKT, and α-tubulin served as controls. (**G**) Representative western blot of p-AMPK and total AMPK after treatment with RES and FFA in HepG2 cells. (**D**), (**F**), and (**H**) Densitometric analysis of the blots shown in (**C**), (**E**), and (**G**), respectively. Data were presented as the mean ± SD, *n* ≥ 3. (∗) *p* < 0.05 and (∗∗) *p* < 0.01, versus control group; (#) *p* < 0.05 and (##) *p* < 0.01, versus FFA group; (△)*p* < 0.05 and (△△)*p* < 0.01, versus RES pretreatment with FFA group.

To further assess the effect of RES on the intracellular redox status of energy metabolism homeostasis, lipid accumulation and expression of related proteins were tested with redox regulator and glutathioneprecursor N-acetylcysteine (NAC) [27]. The Oil Red O staining results indicated that NAC markedly decreased lipid droplet accumulation qualitatively and quantitatively compared with that in the RES-pretreated group (Figure 4A,B). As shown in Figure 4C,D, NAC pretreatment significantly increased ACC phosphorylation by 21.8% and dramatically suppressed the expression levels of FAS, SREBP-1c, and PPARγ by 16%, 14%, and 7%, respectively, compared with the RES-treated group. Meanwhile, NAC efficiently upregulated the phosphorylation of IRS-1 (Tyr 612) and AKT and inhibited the phosphorylation of IRS-1 (Ser 307) (Figure 4E,F). In addition, AMPK phosphorylation was also strongly enhanced by NAC pretreatment (Figure 4G,H).

### 2.4. RES-Modulated Circadian Misalignment in Metabolic Disorders

**Figure 5:**
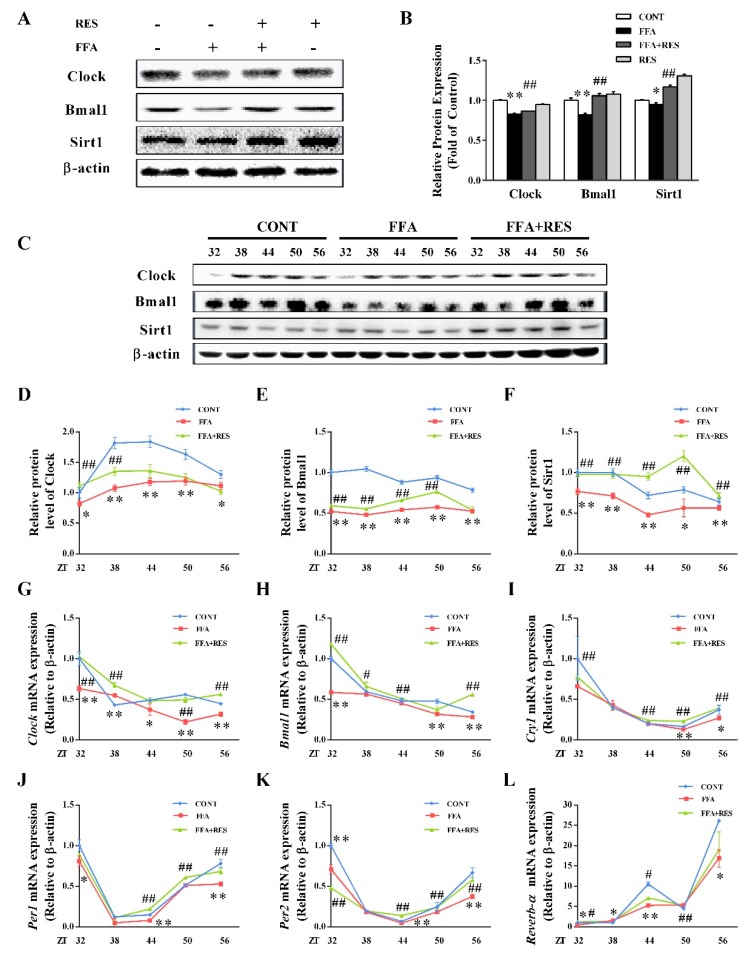
Intervention effect of RES on circadian misalignment under metabolic disorders. HepG2 cells were pretreated with RES (100 µM) for 6 h and with FFA (100 µM) for 24 h. (**A**) Clock, Bmal1 and Sirt1were detected by western blot analysis in HepG2 cells, and β-actin was used as a loading control. (**B**) Densitometric analysis of the blots shown in (**A**). After 2 h serum shock, HepG2 cells were pretreated with RES (100 µM) for 6 h and with FFA (100 µM) with 0.1% BSA for 24 h. Cells were then collected for both mRNA and protein analysis at 6 h intervals between 32 h and 56 h time points. (**C**) The effects of RES on FFA-induced clock gene changes were determined by western blot analysis. Clock, Bmal1, and Sirt1 were detected in HepG2 cells, and β-actin was used as a loading control. (**D**–**F**) Densitometric analysis of the blots shown in (**C**). (**G**–**L**) mRNA levels of circadian oscillator components, namely *Clock*, *Bmal1*, *Cry1*, *Per1*, *Per2* and *Reverb-α* in HepG2 cells. Transcript levels were measured using real-time quantitative PCR (RT-qPCR) and normalized to β-actin mRNA levels. Data were presented as the mean ± SD, *n* ≥ 3. (∗) *p* < 0.05 and (∗∗) *p* < 0.01, versus control group; (#) *p* < 0.05 and (##) *p* < 0.01, versus FFA group.

As presented in Figure 5A,B and Figure S1A,B, the protein levels of Clock and Bmal1 were significantly decreased by FFA compared with control group. However, these levels were substantially reversed by RES. To assess whether RES ameliorated the molecular clock under metabolic disorder conditions, HepG2 cells were exposed to RES (100 µM) for 6 h and then treated with FFA for 24 h after a 2 h serum shock. Next, HepG2 cells were collected for protein and mRNA analysis at 6 h intervals between 32 h and 56 h time points. As shown in Figure 5G–L, Clock, Bmal1, and Clock-related genes (*Cry1*, *Per1*, *Per2*, *Reverb-α*) maintained their rhythmic mRNA expression in the control group. After FFA stimulation, the oscillatory amplitude of core circadian clock components were dramatically weakened in HepG2 cells relative to the control group. FFA also induced the phase shift of Clock and Bmal1 (Figure 5G,H) expression rhythms. Interestingly, RES pretreatment efficiently reverted the relative shallow oscillatory amplitude and phase shift of the circadian clock triggered by FFA. To further investigate the effect of RES on the circadian clock, we verified whether the changes in protein levels were related to the changes in gene expression (Clock, Bmal1, sirtuin 1 (Sirt1)) by western blot analysis. As illustrated in Figure 5C–F, FFA dramatically decreased the protein expression levels of Clock, Bmal1, Sirt1 and led to a phase shift, partially reversed by RES, similar to their transcript levels. Taken together, these data revealed that the downregulation of core components under hepatic metabolic disorders was alleviated by RES.

### 2.5. RES Eliminated FFA-Induced Lipid Metabolism Imbalance by Regulating the Circadian Clock in Hepatic HepG2 Cells

**Figure 6:**
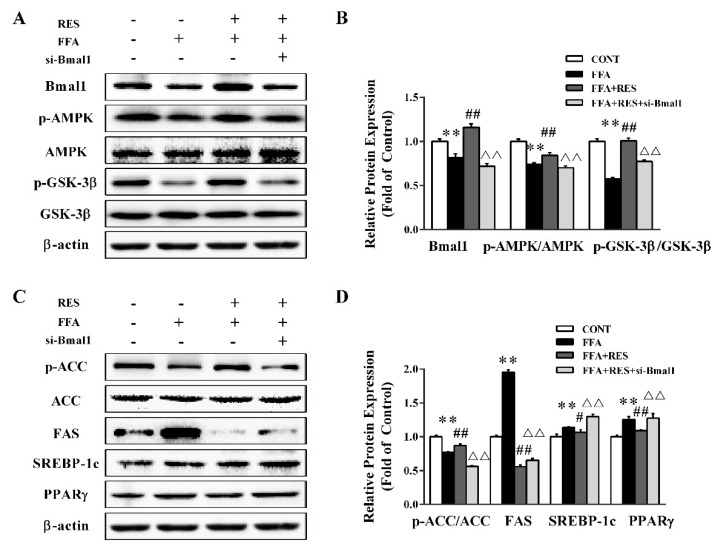
Bmal1 plays a critical role in RES-alleviated lipid metabolism misalignment initiated by FFA exposure in hepatic HepG2 cells. HepG2 cells were exposed to si-Ctrl or si-Bmal1 for 48 h, pretreated with or without RES (100 µM), and treated with FFA (100 µM) with 0.1% BSA for 24 h. (**A**) Representative western blot of Bmal1, p-AMPK, p-GSK3β, total AMPK, and total GSK3β after treatment with RES and FFA in HepG2 cells. (**C**) The expression levels of p-ACC, FAS, SREBP-1c, and PPARγ were detected in cells by western blot analysis. ACC and β-actin served as controls. (**B**) and (**D**) Densitometric analysis of the blots shown in (**A**) and (**C**), respectively. Data were presented as the mean ± SD, *n* ≥ 3. (∗) *p* < 0.05 and (∗∗) *p* < 0.01, versus control group; (#) *p* < 0.05 and (##) *p* < 0.01, versus FFA group; (△) *p* < 0.05 and (△△) *p* < 0.01, versus RES pretreatment with FFA group.

To further evaluate the effect of circadian clock on hepatic glycolipid metabolism homeostasis, small interfering RNAs (siRNAs) were used to knock down Bmal1 in HepG2 cells. As shown in Figure 6A,B, the silencing of Bmal1 expression with siRNA transfection markedly inhibited the Bmal1 protein level by 38.5% relative to that of the RES group. Consistently, Bmall1 knockdown substantially blunted the RES-stimulated phosphorylation of AMPK and GSK-3β (Figure 6A,B). Additionally, RES pretreatment dramatically elicited ACC phosphorylation and reduced the expression levels of FAS, SREBP-1c, and PPARγ compared with those of the FFA treatment group, which were abrogated by the silence of Bmal1 (Figure 6C,D). Taken together, these data demonstrated that RES restored lipid metabolism misalignment in a Bmal1-dependent manner.

## 3. Discussion

Studies have shown that RES inhibits cell viability in a dose- and time-dependent manner, but these effects occur at least 12 h after RES treatment. Marcsek et al. indicated that 0–100 µM RES did not lead to HepG2 cell toxicity after treatment for 24 h [28]. Li et al. demonstrated that treatment with 100 µM RES for 6 h was not toxic to DMEM-pretreated HepG2 cells but was toxic at 24 h and 48 h [29]. Meanwhile, Ou et al. found that 10 µM RES was significantly toxic to HepG2 cells after 48 h treatment [30]. Consistent with previous studies, 6 h RES pretreatment of HepG2 cells had no significant toxic effects on cells. In addition, low doses of RES (10 µM) had no stimulatory effects on HepG2 cells, which was not observed in our study [29].

The mammalian circadian clock consists of a main pacemaker located at SCN and peripheral circadian clock located in other tissues, which coordinate internal physiological processes and circadian rhythms [10,12]. The liver, which is the most important peripheral clock of mammals, plays a fundamental role in modulating fatty acid, glucose, and xenobiotic metabolism [16,31]. Evidence shows that HFD impairs Bmal1 recruitment to target chromatin sites [32]. Chronic circadian rhythm damage caused by HFD-induced obesity erroneously activates several hepatic metabolic pathways, suggesting that core clock protein dysregulation is associated with metabolic disorders in the liver [33]. Epidemiological studies have shown that circadian misalignment is associated with a range of diseases, such as obesity, NAFLD, and IR [34,35]. Oleic acid (OA) (C18:1) and palmitic acid (PA) (C16:0), two of the most abundant fatty acids in the human diet, could significantly disrupt the circadian rhythm and damage biological clock function [21,22,36]. Consistent with the previous study, we found that the control group maintained a relatively good oscillation rhythm, whereas FFA addition down-regulated the expression of Clock, Bmal1, and Clock-related genes and engendered peak ectopic (Figure 5).

Compelling evidence demonstrates that strengthening the circadian rhythm amplitude is one of the nutritional treatments for diseases related to metabolic disorders [37]. Recently, the search for natural nutraceuticals that alleviate metabolic syndrome by regulating circadian rhythms has attracted considerable interest [38]. Food components, such as glucose, sodium, amino acids, caffeine, thiamine, and ethanol, are capable of resetting or phase-shifting circadian rhythms [39,40]. Mi et al. found that EGCG (a biologically active component in green tea) can regulate glucosamine-induced HepG2 hepatocyte IR via the core circadian protein Bmal1 [23]. In *db*/*db* mutant mice, the clock is also obligated for the remitting effects of nobiletin on metabolic disorders [37]. A recent study showed that RES reduced the body weight and systemic metabolic status in HFD-fed C57BL/6J mice and modified the rhythmic expression of clock genes [26]. Moreover, the underlying mechanism of maintaining lipid homeostasis might be mediated by the circadian rhythm. To further explore the causal relationship and mechanism between the circadian clock and lipid metabolism disorders upon RES treatment, 100 µM RES was used to investigate the mechanism of RES against lipid metabolism disorders. The results indicated that RES pretreatment restored the FFA-induced changes in circadian rhythm amplitude and alleviated intracellular lipid metabolism abnormalities in a clock-dependent manner (Figure 5 and Figure 6C), which may provide a new possibility for disease-related nutritional interventions of the circadian clock.

Numerous studies found that HFD could induce liver histological alterations, lipid metabolism disorders, and NAFLD in mice [41,42,43]. Meanwhile, OA and PA are the most abundant fatty acids in our daily diet [42]. Qi et al. showed that PA treatment leads to an imbalance of glucose and lipid metabolism in hepatocytes and stimulates the expression of lipid synthesis-related proteins [22]. Malhi et al. found that OA (C18:1) and PA (C16:1) are lipotoxic to primary hepatocytes, HepG2 cells, Huh7 cells (a human hepatoma cell line), and MRH7777 cells (a rat hepatocellular carcinoma cell line) and induce apoptosis in adipocytes [44]. Concurrently, FFA (OA:PA = 2:1) treatment induces lipid metabolism disorders, and the increased influx of FFAs into the liver is one of the key pathogenic processes [21,45]. Similarly, in this study, FFA treatment led to intracellular lipid droplet accumulation and upregulated the expression of lipid synthesis-related proteins, as well as insulin signaling pathway dysfunction (Figure 1D, Figure 2A, and Figure S1E). A large number of functional food factors are involved in lipid metabolism regulation, such as nobiletin, sesamol, and cichoric acid [21]. NobiletinNOB effectively reversed glucolipid metabolism disorders triggered by PA by increasing the insulin/IGF-1 signaling pathway and regulating key enzymes of *de novo* lipogenesis [22]. Moreover, sesamol ameliorated hepatic lipogenesis elevated by high fat and high fructose diets (HFFD) by inhibiting the expression of *Pparg*, *Srebp1c*, *Fasn*, and *Acaca* genes in the liver [46]. Here, our results revealed that RES remarkably reversed intracellular lipid accumulation and the abnormal expression of lipid synthesis-related proteins caused by FFA (Figure 1D, Figure 2A, and Figure S1E).

Recently, interactions between circadian rhythms and cell metabolism have been demonstrated [47]. Homozygous clock mutant mice, which have metabolic syndromes, such as elevated body weight, hyperlipidemia, hepatic steatosis, and hypolipidemia, are used to investigate the general circadian control of metabolism [18]. Evidence has shown that chronic circadian rhythm damage caused by HFD-induced obesity activates several liver metabolic pathways, revealing that core clock protein dysregulation is related to hepatic metabolic disorders [33]. Our results were consistent with those of previous studies. In the present study, we found significant circadian damages and lipid metabolism disorder after treatment with FFA in HepG2 cells and primary hepatocytes (Figure 5, Figure S1A,B). Consistent with previous studies, we found abnormal levels of lipid synthesis-related proteins after silencing the protein expression of Bmal1 in HepG2 cells, and the beneficial effects of RES pretreatment were counteracted after silencing the expression of Bmal1 (Figure 6). HFD reduced the expression of Bmal1/Clock transcription complex at the mRNA level, indicating the link between the circadian clock and metabolic disorders [43]. Unfortunately, we did not explore the specific mechanisms by which metabolism affects the function of the circadian clock. Thus, further work is needed to find the specific targets.

In summary, the present work indicated that the natural polyphenolic compound RES maintains lipid metabolism homeostasis in a Bmal1-dependent manner. Our research provides crucial evidence for the relevance of circadian clocks in the treatment of metabolic syndrome and NAFLD.

## 4. Materials and Methods

### 4.1. Cell Culture and Treatment

The HepG2 cell line was supplied by the Fourth Military Medical University (Xi’an, Shaanxi, China) and cultured in RPMI -1640 (Hyclone, Logan, UT, USA), 10% fetal bovine serum, and 100 IU/mL penicillin and 100 µg/mL streptomycin at 37 °C in a humidified atmosphere containing 5% CO_2_. RES (purity ≥ 99%) (R5010) was purchased from Sigma-Aldrich (Shanghai, China). A total of 100 µM FFA (OA:PA = 2:1) was prepared as previously described [21]. The cultured cells were pretreated with RES (100 µM) for 6 h and then treated with 100 µM FFA with 0.1% BSA in the medium for 24 h. After treatment, the cells were harvested for further analysis.

### 4.2. Isolation and Treatment of Primary Murine Hepatocytes

Primary cultured mouse hepatocytes were obtained from C57BL/6J mice by collagenase perfusion as previously described [46]. All experimental procedures were in accordance with the Guide for the Care and Use of Laboratory Animals: Eighth Edition, ISBN-10: 0-309-15396-4. The animal protocol was approved by the Animal Ethics Committee of Xi’an Jiaotong University. Hepatocytes were pretreated with RES (100 µM) for 6 h. Subsequently, they were treated with FFA (100 µM) with 0.1% BSA in culture medium for 24 h and collected for western blot analysis.

### 4.3. Small Interfering RNA (siRNA) Transfection of Hepatocytes

Hepatocytes were transfected with si-Ctrl or si-Bmal1 using Lipofectamine 2000 (Invitrogen). After transfection for 48 h, the cultured HepG2 cells were pretreated with RES (100 µM) for 6 h. Then, they were treated with FFA (100 µM) with 0.1% BSA in the culture medium for 24 h. Following the treatment, the cells were collected for further analysis. Afterward, the cells were harvested for further detection.

### 4.4. Synchronization of HepG2 Cells

Cells were shocked with 50% horse serum for 2 h and pretreated with RES (100 µM) for 6 h. Then, they were treated with 100 µM FFA with 0.1% BSA in culture medium for 24 h and harvested for mRNA and protein detection at 6 h intervals between 32 h and 56 h time points.

### 4.5. Cell Viability

HepG2 cells were subcultured at a density of 2 × 10^4^ cells/mL in a 96-well plate and incubated overnight at 37 °C with 5% (*v*/*v*) CO_2_. After removing 100 µL of medium from each well, the HepG2cells were treated with or without RES (100 µM) for 6 h and then treated with or without FFA (100 µM) for 24 h after washing with PBS as previously described [48].

### 4.6. Lipid Levels of Hepatocytes

Hepatocytes were pretreated with RES (100 µM) for 6 h. Then, they were treated with FFA (100 µM) with 0.1% BSA in the culture medium for 24 h and then harvested for further analysis. The contents of total cholesterol (T-CHO, A111-2), triglycerides (TG, A110-1), and the activity of catalase (CAT, A007-1-1) were measured using enzymatic assay kits (Nanjing Jiancheng Bioengineering Institute, Nanjing, China). The amount of protein in the supernatant was measured using a bicinchoninic acid kit (Thermo Fisher, Waltham, MA, USA). Oil Red O staining was used as described previously [49]. After treatments, TG were visualized by light phase contrast microscopy and quantitative determination was obtained by measuring the absorbance of cell monolayers at 492 nm in a spectrophotometer (Ultramark, Biorad).

### 4.7. Mitochondrial Membrane Potential (MMP) Assay

The mitochondrial membrane potential disruption was measured using the mitochondrial membrane potential assay kit with a JC-1 probe (Beyotime, China). Cells were subcultured in a 96-well plate at a density of 2 × 10^4^ cells/mL and incubated overnight at 37 °C with 5% (*v*/*v*) CO_2_, as described previously [50].

### 4.8. Detection of Reactive Oxygen Species (ROS)

ROS production was measured using a 2′,7′-dichlorodihydrofluorescein diacetate (DCFH-DA) assay kit (Beyotime, China). Cells were washed with PBS twice (pH 7.4). The DCF fluorescence intensity of the supernatant was measured using a fluorescence microplate reader at 485 nm excitation and 535 nm emission, as described previously [51]. The amount of protein in supernatant was measured using bicinchoninic acid (BCA) kit (Thermo Fisher, USA).

### 4.9. RNA Preparation and Quality Control and Real-Time Quantitative PCR (RT-qPCR)

Total RNA was extracted from cells using an RNA extraction kit (TaKaRa, MiniBEST Universal RNA Extraction Kit, Dalian, China) as previously described [51]. RNA purity and integrity were evaluated using the Quawell 5000 UV-Vis spectrophotometer (Quawell Technology, San Jose, CA, USA).

Total RNA (500 ng) was reverse-transcribed into cDNA using the PrimeScript RT Master Mix reverse transcription kit (TaKaRa PrimeScript RT Master Mix, Dalian, China), and mRNA expression was quantified by RT-qPCR using a SYBR Green PCR kit (TaKaRa SYBR Premix Ex Taq II, Dalian, China) and CFX96 real-time system (Bio-Rad, Hercules, CA). Gene-specific mouse primers were used as shown in Table 1. Ct values were normalized to β-actin, and the relative gene expression was calculated with the 2^−△△Ct^ method.

### 4.10. Western Blot

The treated HepG2 cells and primary hepatocytes were lysed by Cell Lysis Buffer (Beyotime Institute of Biotechnology, Jiangsu, China) and the total protein concentration was determined by a BCA protein kit (Thermo Fisher, Shanghai, China). SDS buffer was added to the sample and the protein was denatured in boiling water for 5 min. Sample proteins were separated by SDS-PAGE and transferred to PVDF membranes. Protein bands are visualized with enhanced chemiluminescence reagents using appropriate antibodies. Antibodies against Clock (ab93804) and Bmal1 (ab93806) were purchased from Abcam (Abcam, Cambridge, MA). Antibodies against mitochondrial respiratory chain complex I (NADPH-diaphorase, SC-20493) and III (cytochrome reductase, SC-69064), IRS-1 (SC-559), α-tubulin (SC-5286), PPARγ (H-100, sc-7196), SREBP-1c (C-20, sc-366), and β-actin (I-19) were purchased from Santa Cruz Biotechnology (Santa Cruz, USA). Antibodies against p-IRS-1 (Ser 307, 2384), AKT (4060), p-AKT (Ser 473, 8242), AMPK (23A3, 2603), p-AMPK (Τhr172, 2535), GSK-3β (123456), p-GSK-3β (ser9, 9336), Sirt1 (1F3, 8469), ACC (3662), p-ACC (Ser 79, 11818), and FAS (4C3, 8023) were purchased from Cell Signaling Technology Company (Shanghai, China). The p-IRS-1 (Tyr 612) antibody was purchased from Millipore (Bedford, MA, USA). Densitometric analysis of western blot was performed with Quantity One 4.6.2 software (Bio-Rad, Hercules, CA, USA).

### 4.11. Statistical Analysis

Data were reported as the mean ± SD from at least three independent experiments. Significant differences between measurements for the control and treated samples were analyzed using one-way factorial analysis of variance, followed by Tukey’s test (SPSS 16.0). Means were considered to be statistically significant if *p* < 0.05.

## Figures and Tables

**Table 1:** Primer sequences used for quantitative RT-PCR analysis.

	Forward Primer	Reverse Primer
*Clock*	AAAATACTCTCTACTCATCTGCTGG	ATGGCTCCTTTGGGTCTATTG
*Bmal1*	CTGGCTAGAGTGTATACGTTTGG	GGTCACCTCAAAGCGATTTTC
*Cry1*	TTACACTATGCTCATGGCGAC	GTGCTCTGTCTCTGGACTTTAG
*Per1*	ATTCCGCCTAACCCCGTATGTGACC	GTGTGCCGCGTAGTGAAAATCCTCTTGT
*Per2*	CCCTTCCGCATGACGCCCTACCTG	GACCGCCCTTTCATCCACATCCTG
*Reverb-αα*	CATGGTGCTACTGTGTAAGGTGTGT	CACAGGCGTGCACTCCATAG
*β-actin*	TCCACCTTCCAGCAGATGTG	GCATTTGCGGTGGACGAT

## Supplementary Materials

The following are available online, Figure S1.

## Abbreviations

ACC, acetyl-CoA carboxylase; AKT, protein kinase B; AMPK, AMP-activated protein kinase; Bmal1, brain and muscle Arnt-like protein-1; CAT, catalase; Clock, circadian locomotor output cycles kaput; Cry, cryptochromes; FAS, fatty acid synthase; FFA, free fatty acid; GSK-3β, glycogen synthase kinase β; HFD, high-fat diet; HFFD, high fat and high fructose die; IR, insulin resistance; IRS-1, insulin receptor substrate 1; MMP, mitochondrial membrane potential; MTT, 3-(4,5-dimethyl-2-thiazolyl)-2,5-diphenyl-2-H-tetrazolium bromide; NAFLD, nonalcoholic fatty liver disease; OA, oleic acid; PA, palmitic acid; p-ACC, phospho-acetyl-CoA carboxylase; Per, period; PPARγ, peroxisome proliferator activated receptor gamma; RES, resveratrol; ROS, reactive oxygen species; RT-qPCR, real-time quantitative PCR; SCN, suprachiasmatic nuclei; Sirt1, sirtuin 1; SREBP-1c, sterol regulatory element-binding protein 1c; T-CHO, total cholesterol; TG, triglyceride.
