# Supplementary material for: Resveratrol Maintains Lipid Metabolism Homeostasis via One of the Mechanisms Associated with the Key Circadian Regulator Bmal1
Source: Molecules. 2019 Aug 12;24(16):2916. doi: 10.3390/molecules24162916 (PMC6718980; doi:10.3390/molecules24162916)
Supplement: Supplementary file 1 [file molecules-24-02916-s001.pdf]

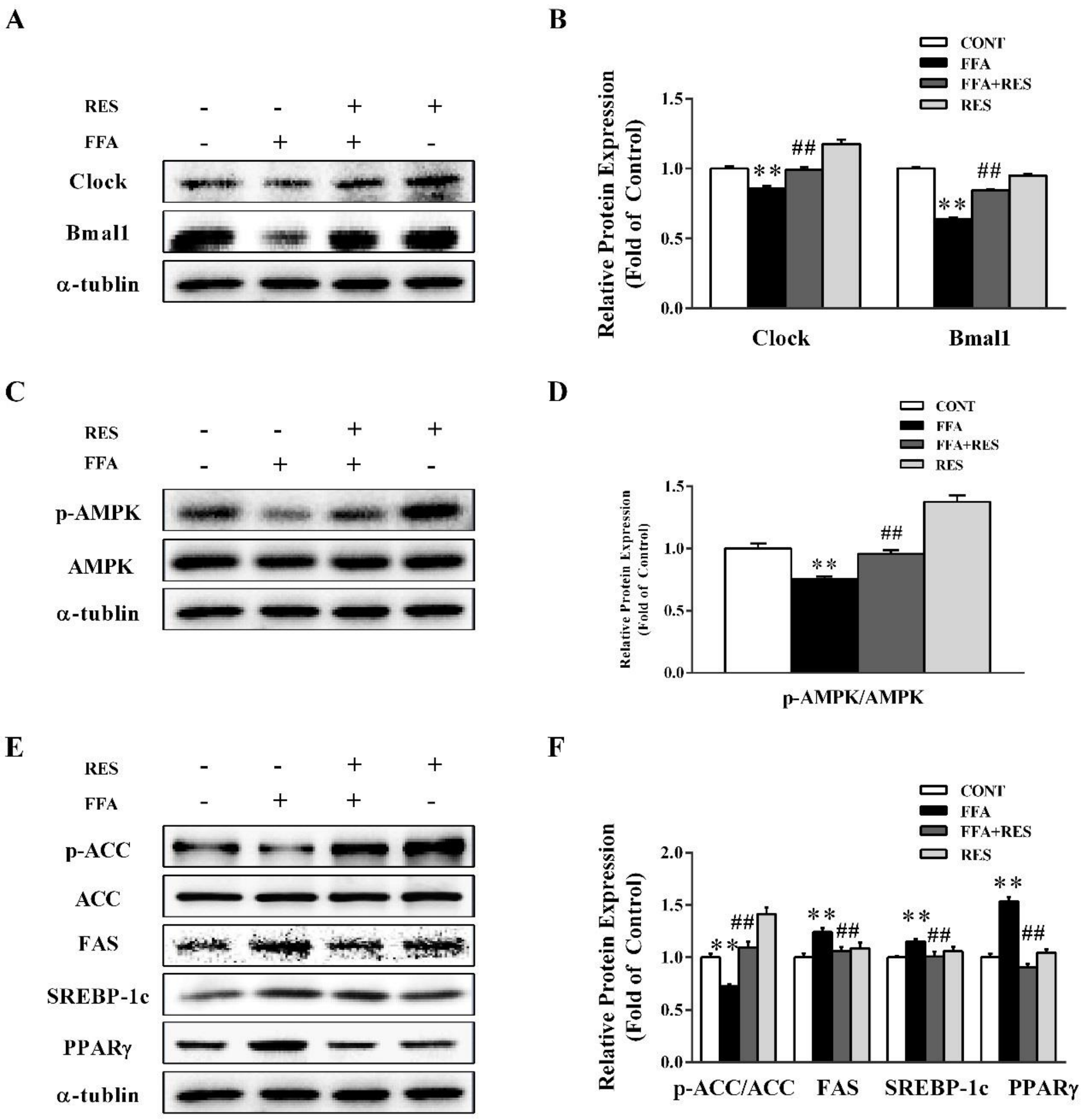

Figure S1. RES attenuated FFA-triggered hepatic lipid metabolism unbalance in primary hepatocytes. Primary hepatocytes were pretreated with or without RES (100 μM) and treated with FFA (100 μM) with 0.1% BSA for 24 h. (A) and (C) The expression levels of Clock, Bmal1, p-AMPK, and total AMPK were detected in cells, and α-tubulin was used as a loading control. (E) Representative Western blots of p-ACC, FAS, SREBP-1c, and PPARγ after treatment with RES and FFA in primary hepatocytes. (B), (D), and (F) Densitometric analysis of the blots shown in (A), (C), and (E). Data were presented as the mean ± SD, $n \geq 3$. (*) $p < 0.05$ and (**) $p < 0.01$, versus control group; (#) $p < 0.05$ and (##) $p < 0.01$, versus FFA group.
